# Associations of educational attainment, occupation and community poverty with knee osteoarthritis in the Johnston County (North Carolina) osteoarthritis project

**DOI:** 10.1186/ar3492

**Published:** 2011-10-19

**Authors:** Leigh F Callahan, Rebecca J Cleveland, Jack Shreffler, Todd A Schwartz, Britta Schoster, Randy Randolph, Jordan B Renner, Joanne M Jordan

**Affiliations:** 1 Thurston Arthritis Research Center, Departments of Medicine and Social Medicine, 3300 Thurston Building, CB # 7280, University of North Carolina, Chapel Hill, NC 27599-7330, USA; 2 Department of Biostatistics, Gillings School of Global Public Health, University of North Carolina, 3106E McGavran-Greenberg Hall, CB #7420, Chapel Hill, NC 27599-7420, USA; 3 Sheps Center for Health Services Research, 725 Martin L King Jr Blvd, Campus Box 7590, University of North Carolina, Chapel Hill, NC 27599-7590, USA; 4 Department of Radiology, University of North Carolina, 509 Old Infirmary Bldg, Campus Box 7510, Chapel Hill, NC 27599, USA

**Keywords:** knee osteoarthritis, educational attainment, occupation, community poverty, socioeconomic status

## Abstract

**Introduction:**

The purpose of this study was to examine data from the Johnston County Osteoarthritis (OA) Project for independent associations of educational attainment, occupation and community poverty with tibiofemoral knee OA.

**Methods:**

A cross-sectional analysis was conducted on 3,591 individuals (66% Caucasian and 34% African American). Educational attainment (< 12 years or ≥12 years), occupation (non-managerial or not), and Census block group household poverty rate (< 12%, 12 to 25%, > 25%) were examined separately and together in logistic models adjusting for covariates of age, gender, race, body mass index (BMI), smoking, knee injury and occupational activity score. Outcomes were presence of radiographic knee OA (rOA), symptomatic knee OA (sxOA), bilateral rOA and bilateral sxOA.

**Results:**

When all three socioeconomic status (SES) variables were analyzed simultaneously, low educational attainment was significantly associated with rOA (odds ratio (OR) = 1.44, 95% confidence interval (CI) 1.20, 1.73), bilateral rOA (OR = 1.43, 95% CI 1.13, 1.81), and sxOA (OR = 1.66, 95% CI 1.34, 2.06), after adjusting for covariates. Independently, living in a community of high household poverty rate was associated with rOA (OR = 1.83, 95% CI 1.43, 2.36), bilateral rOA (OR = 1.56, 95% CI 1.12, 2.16), and sxOA (OR = 1.36, 95% CI 1.00, 1.83). Occupation had no significant independent association beyond educational attainment and community poverty.

**Conclusions:**

Both educational attainment and community SES were independently associated with knee OA after adjusting for primary risk factors for knee OA.

## Introduction

The most common form of arthritis is osteoarthritis (OA), with more than 27 million Americans having some form of OA [1]. Knee OA and hip OA are the two types of OA considered to have the largest impact as a result of their effects on mobility [2]. Risk factors for knee OA include advanced age, female gender, high body mass index (BMI], prior knee injury, positive family history of OA and lower levels of socioeconomic status [1,3-6].

Associations between lower levels of educational attainment and both OA presence and poorer health status outcomes have been noted in a number of studies [4,6-12]. Only two of these studies, however, examined radiographic OA [4,6]. Using data collected from 1971 to 1975, Hannan and colleagues evaluated the first National Health and Nutrition Examination Survey (NHANES-I) and found that low educational attainment was associated with both radiographic and symptomatic knee OA, but only the associations with symptomatic knee OA remained independent of occupation, age, race, obesity and knee injury [6]. Our group examined limited educational attainment and radiographic and symptomatic knee OA in a more contemporary cross-sectional sample of 2,627 non-Hispanic African American and Caucasian adults aged 45 years and older from the Johnston County OA Project [4]. We found that after adjustment for known risk factors, educational attainment was associated with increased presence of symptomatic knee OA in both men and women and with radiographic knee OA in women [4].

In other chronic diseases and in self-reported arthritis, the poverty rate of one's community has been found to be associated with disease incidence and prevalence independent of a person's level of educational attainment [13-18]. In Canada, two studies have examined associations between provincial or regional context with the prevalence of self-reported arthritis [16,17]. In the study examining inter-provincial variations after adjusting for individual level SES measures, and other important risk factors, arthritis and most chronic conditions were reported less frequently in Quebec compared to other provinces in Canada [16]. Another study from Canada found that differences in the prevalence of self-reported arthritis were associated with both individual level SES measures and regional level measures, such as the proportion of families in a region with low incomes [17]. In the only US study examining area-level measures and arthritis prevalence, 7,770 US participants who were followed in a family medicine practice-based research network were evaluated. In that population, both non-Hispanic African American and Caucasian participants living in communities with high rates of poverty showed increased odds of self-reported arthritis after adjusting for individual educational attainment [12].

To date, such community contextual factors have not been evaluated in radiographically defined knee OA. The purpose of this study is to examine associations between educational attainment, occupation (individual level SES), and neighborhood household poverty rate (community level SES) with radiographic knee OA and symptomatic knee OA in the Johnston County OA Project. We hypothesized that both individual and community level SES would be independently associated with radiographic and symptomatic knee OA.

## Materials and methods

**Figure 1:**
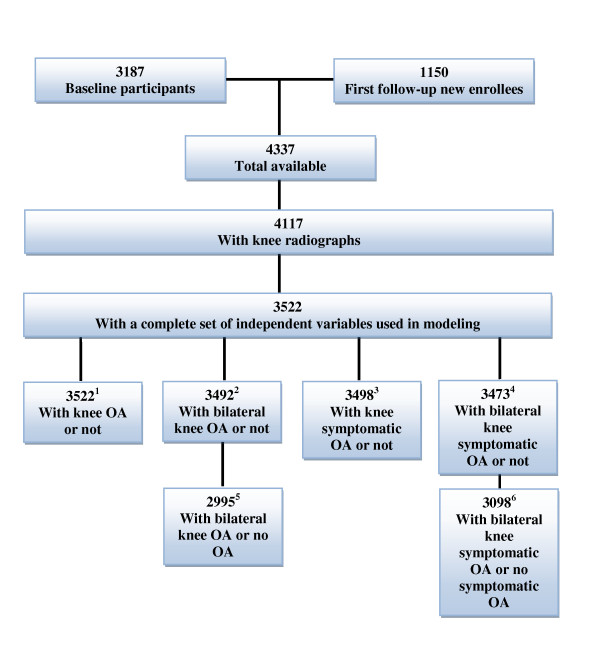
**Participant flow chart**. ^1^Sample excludes those without radiographic data for at least one knee (as used in Table 1 and modeling); ^2^sample excludes those without radiographic data for both knees (as used in Table 1); ^3^sample excludes those without radiographic and pain data for at least one knee (as used in Table 1 and modeling); ^4^sample excludes those without radiographic and pain data for both knees (as used in Table 1); ^5^sample excludes those with unilateral knee OA (as used in modeling); ^6^sample excludes those with unilateral symptomatic knee OA (as used in modeling)

This cross-sectional study uses baseline data derived from 4,337 individuals who entered the Johnston County OA Project in two enrollment periods, 1991-1997 (original baseline) and 2003-2004 (cohort enrichment baseline for new enrollees). The final study cohort numbered 4,117 for people with information for at least one knee outcome (Figure 1). The Johnston County OA Project is an ongoing, population-based study of OA which is described in detail in a previous publication [5]. All participants were living in Johnston County, North Carolina at the time of enrollment. Participants were civilian, non-institutionalized non-Hispanic African Americans and Caucasians 45 years of age or older who were physically and mentally capable of completing the protocol involving radiographs, home interview, and physical examination. Two home interviewer-administered interviews were completed on all participants. Participants also underwent a limited clinical and functional examination, including assessment of weight (kg) using a balance beam scale, height (cm) measured with a stadiometer, and radiographic examination of the knees. The current study is a cross-sectional evaluation intended to examine the presence of radiographic knee OA and symptomatic knee OA and their associations with individual level SES factors (educational attainment and occupation) and a community contextual factor (Census-based household poverty rate), while adjusting for important individual covariates. This study was approved by the Institutional Review Boards of the University of North Carolina Schools of Medicine and Public Health and the Centers for Disease Control and Prevention. All participants gave written informed consent at the time of recruitment.

### Primary outcome measures

Data from participants' interviews and examinations in this large population screening were used to define a sample with the following four outcomes: radiographic tibiofemoral knee OA and a subgroup of this sample with symptomatic tibiofemoral knee OA, defined either generally (one or both knees) or bilaterally (both knees). We used both radiographic and symptomatic definitions since they are the two most commonly used.

### Radiographic knee OA

X-rays of the knee in the baseline enrollees (1991 to 1997) were anterior-posterior (AP) whereas X-rays on the new enrollees (2003 to 2004) were posterior-anterior (PA). A large subset (*n *= 1,627) of baseline participants were followed up in the 1999 to 2003 period and had both AP and PA X-rays. Agreement between the AP and PA as to the presence or absence of radiographic OA by Kellgren-Lawrence grade (KL grade) was 89.2% (AP(yes)/PA(no) in 4.9% of cases and AP(no)/PA(yes) in 5.9% of cases) with κ 0.73, 95% confidence interval (CI) 0.69 to 0.76 [18]. Since results were so similar, the determinations of radiographic OA from the two methods of X-ray were deemed comparable for this study.

Based on X-ray readings, the KL-grade of each knee was assigned on a scale of 0 to 4 by a single bone and joint radiologist (JBR), where higher values show stronger evidence of osteoarthritic deterioration of the joint. A diagnosis of radiographic knee OA was indicated from a KL-grade of 2 or higher in at least one knee. Bilateral radiographic knee OA was defined as having KL grade ≥ 2 in both knees.

### Symptomatic knee OA

The presence of symptomatic knee OA was defined as at least one knee with radiographic OA and reported symptoms (pain, aching, or stiffness on most days) of any intensity in the same knee. Bilateral symptomatic knee OA was defined as radiographic OA and symptoms in both knees.

### Primary exposures of interest

The primary exposures of interest included three measures of SES: two individual level measures (educational attainment and occupation) and a contextual community level measure (Census-based household poverty rate).

#### Individual level measures of SES

Educational Attainment: Education is represented as an indicator for less than 12 years of formal schooling or at least 12 years of schooling (referent).

Occupational Category: Occupation, based on the job held for the longest duration, was represented by an indicator variable that distinguished between occupations that are non-managerial and those that are managerial/professional (referent), although it may not be a perfect representation. There are six U.S. Census classifications applicable to the principal occupation cited by the participants. The non-managerial occupations were (1) Service; (2) Farming, Forestry, and Fishing; (3) Precision Production, Craft, and Repair; (4) Operators, Fabricators, and Laborers. The managerial were (5) Managerial and Professional; (6) Technical, Sales, and Administrative Support [19].

#### Community level measure of SES

Household Poverty Rate: The percentage of households with income below the poverty level in a US Census block group is the household poverty rate variable. Block groups generally contain between 600 and 3,000 people. Each participant's physical address was geocoded, linked to the block group identification number, and then used to extract aggregated Census information. The address at enrollment was linked to the most recent Census information at the time of enrollment, 1990 for baseline enrollees and 2000 for new enrollees from the cohort enrichment. The Census-based household poverty rate for a participant's block group is intended to indicate the contextual, community SES where the resident lives. Two residents of the same block group will share the same household poverty rate, but may have different individual characteristics, such as educational attainment and occupation. The average household poverty rate for the sample was 19.6%. With the intent to compare people residing approximately in the upper and lower quartiles of community poverty, the participants were divided into three groups using cut points of 12% and 25% for their block group poverty rates. This scheme divides participants into three groups of low (referent, 22%), medium (56%) and high (22%) community poverty rates. The household poverty rates for the 67 block groups in the sample range from 0 to 50%.

### Covariates

Associations between SES variables and OA outcomes were adjusted for age, gender, race, BMI, current smoking status, knee injury and occupational activity score. The minimum age for the study was 45 years, and age was expressed in categories as 45 to 54 (referent), 55 to 64, and 65 or older using indicators in the models. For gender, an indicator was used for female versus male (referent). The race variable was an indicator for African American versus Caucasian (referent). Obesity was defined as BMI ≥ 30 (weight in kg/height^2 ^in m^2^). The smoking variable was an indicator of current smoking of tobacco products (with "no" as referent), and knee injury (with "no" as referent) is an indicator of any self-reported previous knee injury. Occupational activity was defined based on self-reports of frequencies of four activities at work: squatting, standing, lifting and walking (0 = never, 1 = seldom, 2 = sometimes, 3 = often, 4 = always). The sum (0 to 16) of the individual occupational activity scores was dichotomized as low (≤9 being referent) or high (≥ 10), based on the median value from the sample. The activity indicator carries direct information about physical stress on the job as opposed to the SES of occupational category, although the two variables are correlated.

### Statistical analysis

Data were prepared and processed into final form using SAS v9.1 (SAS Institute Inc., Cary, NC, USA). The dataset was then converted to STATA v9 (StatCorp LP, College Station, TX, USA) for all analyses. Each person's physical address was geocoded into values of latitude and longitude and assigned to one of the 68 block groups in Johnston County. The Census block group characteristic of household poverty rate was then used as a contextual variable applicable to each participant.

Outcome variables for radiographic OA and symptomatic OA were dichotomous (yes = 1 or no = 0), and logistic regression modeling was utilized. Radiographic OA was considered as present if there was OA in one or both knees and as absent if OA in neither knee. Radiographic bilateral OA was considered as present if there was OA in both knees, and as absent if OA in neither knee, and excluded from analysis if OA in only one knee. Parallel definitions apply to symptomatic OA.

In deciding on the final modeling approach, we considered whether race modified any of the main SES effects for each outcome. In preliminary analyses of all outcomes, we explored every possible model with single SES variables, including race and the race by SES interaction, adjusted for all other covariates. There were no significant interactions at the 0.05 level. Based on these results, race was treated as a covariate rather than stratifying statistical models by race. Similar analyses found no effect modification by gender.

We also examined the percentage of African American residents in the block group as a possible community variable indicating segregation. For the 67 block groups, the percentage of African American residents ranged from 0 to 93% and averaged 16.8%. Because of racial disparities in income, there was a strong positive correlation (0.83) between household poverty rate and this measure of segregation. Ultimately, the percentage of African Americans in the block group was determined to have minor independent predictive value and was dropped from the final modeling approach.

Analyses were carried out on a complete-case dataset where all subjects had no missing values for any covariates. However, owing to the moderate amounts of missing data in the cohort, primarily for occupation and occupational activity (both missing 6.3% of values), multiple imputation of missing observations was performed to evaluate whether complete-case analyses introduced bias. Since it is difficult to verify that variables are missing completely at random (MCAR), our data were considered to be missing at random (MAR) and were assumed to have only a minor impact on the estimates [20]. We performed this imputation for missing covariates using the method of multiple imputation by chained equations (MICE) via IVEware software Version 0.2 (University of Michigan, Ann Arbor, MI, USA). Results using 10 iterations indicate that there are no meaningful differences in findings from data including imputed values compared to the complete case analysis. Therefore, results from a complete-case analysis are reported here.

As we carried out a cross-sectional study on combined data from the original baseline data, which was produced from complex probability sampling, and the enrichment cohort, which was not, we wanted to verify that clustering by the primary sampling unit (PSU) did not affect the estimates or their precision. Analyses accounting for within-PSU correlation using generalized mixed models in the original baseline study. We found no meaningful differences in results accounting for the PSU; therefore, reported analyses do not involve adjustment for PSU.

## Results

The characteristics of the complete sample with all non-missing explanatory variables (*n *= 3,522) are displayed in Table 1. The sample sizes are given in Figure 1, and the percentages for the OA definitions are based on all participants (unilateral OA included). The mean age was 60.9 ± 10.5; 61.7% were female; 39.8% had BMI of at least 30; 21.6% were current smokers of tobacco; 19.1% reported a past knee injury; and 46.9% reported frequent occupational activities (lifting, standing, squatting, walking). In terms of our main variables of interest, SES variables, 33.7% had low educational attainment (less than 12 years), 56.2% had non-managerial occupations at their longest job, and 22.4% lived in block groups with household poverty rates over 25%. Radiographic knee OA was noted in 29.5% of the sample, with 14.7% having bilateral radiographic knee OA, 17.7% with symptomatic knee OA, and 6.3% with bilateral symptomatic knee OA.

**Table 1:** Characteristics of the sample overall

Variable	Total group (*n *= 3,522)
Age group, %	
45 to 54	35.6
55 to 64	29.6
> 64	34.9
Female, %	61.7
BMI ≥ 30%	39.8
African American	33.8
Smoking now, %	21.6
Knee injury, %	19.1
^1^High occupational activity, %	46.9
Educational attainment, %	
Less than 12 yr	33.7
^2^Non-managerial Occupation, %	56.2
Poverty rate group, %	
Low (< 12%)	21.7
Medium (12 to 25%)	55.9
High (> 25%)	22.4
Knee OA, %	29.5
Bilateral Knee OA, %	14.7
Knee Symptomatic OA, %	17.7
Bilateral Knee Symptomatic OA, %	6.3

^1 ^Self-report of above-median frequency for job-related squatting, lifting, standing, and walking.

^2 ^An indicator of lower SES occupational category which may include jobs where one has less control over the pace of their work and tend to exclude jobs described as office work, managerial, professional, technical, and sales.

The odds ratios for the unadjusted associations between the three SES measures with each of the covariates with the four knee outcome variables are displayed in Table 2. Low educational attainment, a non-managerial occupation, and living in a neighborhood with higher rates of household poverty were all significantly (*P *< 0.05) associated with radiographic, bilateral radiographic and symptomatic OA (ORs ranging from 1.39 to 2.29). In addition, low educational attainment (OR = 1.89, 95% CI 1.43, 2.49) and a non-managerial occupation (OR = 1.43, CI 1.08, 1.90) were both significantly associated with bilateral symptomatic OA. The adjusted analyses for each of the SES variables considered in separate models are also shown in Table 2. The magnitudes of the ORs for the SES variables were attenuated when compared to the bivariate results, but each SES variable remained statistically significant for radiographic, bilateral radiographic, and symptomatic knee OA (association of non-managerial occupation and bilateral radiographic knee OA was of borderline significance, OR = 1.24, 95% CI 0.99, 1.55, *P *= 0.056). Those with low educational attainment were 50% more likely than those with higher educational attainment to have radiographic knee OA and bilateral radiographic knee OA and 75% more likely to have symptomatic knee OA. Those living in high poverty areas were almost twice as likely as those not living in such areas to have radiographic knee OA, 64% more likely to have bilateral radiographic knee OA, and 44% more likely to have symptomatic knee OA. Compared to those in managerial occupations, those with non-managerial occupations were about 25% more likely to have radiographic knee OA and bilateral radiographic knee OA, and 33% more likely to have symptomatic knee OA. None of the SES variables were significantly associated with bilateral symptomatic OA, although associations were in the expected direction, and the association between low educational attainment and bilateral symptomatic knee OA was of borderline significance (OR = 1.32, 95% CI 0.97, 1.79, *P *= 0.08). The low frequency of bilateral symptomatic knee OA is likely responsible for the lack of statistical significance.

**Table 2:** Associations between each independent SES variable analyzed singly with each OA outcome.

Independent variables	Knee radiographic OA OR, 95% CI	Bilateral Knee radiographic OA OR, 95% CI	Symptomatic OA OR, 95% CI	Bilateral Symptomatic OA OR, 95%CI
Unadjusted odds ratios for each independent variable singly as associated with each outcome				
Low educational attainment	2.07 (1.78, 2.41)**	2.29 (1.89, 2.78)**	2.23 (1.87, 2.66)**	1.89 (1.43, 2.49)**
Non-managerial occupation	1.39 (1.20, 1.61)**	1.53 (1.25, 1.86)**	1.49 (1.24, 1.78)**	1.43 (1.08, 1.90)*
Medium poverty rate, 12 to 25%	1.24 (1.03, 1.51)*	1.07 (0.83, 1.38)	1.28 (1.01, 1.62)*	1.14 (0.80, 1.63)
High poverty rate, > 25%	1.91 (1.54, 2.38)**	1.85 (1.40, 2.45)**	1.63 (1.25, 2.12)**	1.39 (0.92, 2.09)
**Adjusted odds ratios for each independent variable singly as associated with each outcome**				

Low educational attainment	1.52 (1.28, 1.80)**	1.49 (1.20, 1.86)**	1.74 (1.42, 2.12)**	1.32 (0.97, 1.79)
Non-managerial occupation	1.26 (1.07, 1.48)**	1.24 (0.99, 1.55)	1.33 (1.09, 1.63)**	1.20 (0.88, 1.64)
Medium poverty rate, 12 to 25%	1.24 (1.01, 1.53)*	1.02 (0.78, 1.34)	1.23 (0.96, 1.57)	1.04 (0.71, 1.51)
High poverty rate, > 25%	1.92 (1.50, 2.46)**	1.64 (1.19, 2.27)**	1.44 (1.07, 1.94)*	1.15 (0.73, 1.82)

**^§^**Adjusted for age, gender, race, BMI, smoking, knee injury, and occupational activity score

* *P *< 0.05; ** *P *< 0.01: from test for OR = 1.

^1 ^This table presents odds ratios and 95% confidence interval results of 24 logistic models (3 single SES variables, 4 outcomes, both unadjusted and adjusted).

The results of the fully adjusted logistic model, containing all three SES variables simultaneously, and adjusted for all covariates, are displayed in Table 3. Both low educational attainment (OR = 1.44) and high community poverty rate (OR = 1.83) were independently and significantly associated with radiographic OA. Those with low educational attainment were 44% more likely to have radiographic OA after adjusting for occupation, community poverty and covariates. And, individuals living in a community with a high poverty rate were 83% more likely to have radiographic OA after adjusting for educational attainment and occupation. Both low educational attainment and living in a community with a high poverty rate were independently associated with bilateral knee symptomatic OA as well. Compared to individuals with at least 12 years of schooling, those with low educational attainment were 43% more likely to have bilateral radiographic knee OA and 66% more likely to have symptomatic knee OA after adjusting for occupation, community poverty and other covariates. And, those individuals living in a community with a high poverty rate were 56% more likely to have bilateral radiographic OA and 36% more likely to have symptomatic OA compared to those in communities with low poverty rates, after adjusting for education, occupation and other covariates. Having a non-managerial occupation was not associated with any outcome when adjustments were made for educational attainment and community poverty, in addition to the covariates.

**Table 3:** Associations between all three SES variables analyzed simultaneously with OA outcomes

Variables	Knee radiographic OA OR, 95% CI	Bilateral knee radiographic OA OR, 95% CI	Knee symptomatic OA OR, 95% CI	Bilateral knee symptomatic OA OR, 95% CI
Low educational attainment	1.44 (1.20, 1.73)**	1.43 (1.13, 1.81)**	1.66 (1.34, 2.06)**	1.27 (0.91, 1.76)
Non-managerial occupation	1.07 (0.90, 1.28)	1.07 (0.84, 1.36)	1.09 (0.88, 1.36)	1.10 (0.79, 1.54)
Medium poverty rate, 12 to 25%	1.21 (0.98, 1.48)	0.99 (0.75, 1.29)	1.18 (0.92, 1.51)	1.01 (0.70, 1.48)
High poverty rate, > 25%	1.83 (1.43, 2.36)**	1.56 (1.12, 2.16)**	1.36 (1.00, 1.83)*	1.12 (0.71, 1.76)
Age (55 to 64)	1.68 (1.36, 2.07)**	2.14 (1.59, 2.90)**	1.96 (1.50, 2.54)**	2.34 (1.53, 3.58)**
Age (65 and above)	3.70 (3.00, 4.57)**	5.59 (4.17, 7.50)**	3.69 (2.84, 4.79)**	4.80 (3.17, 7.26)**
Female	1.06 (0.89, 1.25)	1.14 (0.91, 1.42)	1.24 (1.01, 1.52)*	1.58 (1.14, 2.19)**
African American	0.92 (0.77, 1.11)	1.13 (0.89, 1.45)	0.95 (0.77, 1.19)	1.00 (0.72, 1.40)
BMI ≥ 30	2.39 (2.03, 2.81)**	2.89 (2.32, 3.58)**	2.92 (2.40, 3.56)**	3.93 (2.89, 5.35)**
Current smoker	0.68 (0.55, 0.84)**	0.61 (0.45, 0.83)**	0.80 (0.62, 1.04)	0.82 (0.54, 1.25)
Knee injury	2.11 (1.75, 2.55)**	1.90 (1.48, 2.44)**	3.15 (2.55, 3.88)**	2.23 (1.61, 3.08)**
High activity score	1.00 (0.85, 1.17)	1.00 (0.81, 1.24)	1.16 (0.96, 1.41)	1.19 (0.88, 1.59)

**^§^**Age, gender, race, BMI, smoking, knee injury, and occupational activity score

* *P *< 0.05; ** *P *< 0.01: from test for OR = 1.

^1 ^This table presents odds ratios and 95% confidence interval results of four logistic models (four outcomes).

## Discussion

Both low levels of educational attainment and living in a community with a household poverty rate greater than 25% were independently associated with radiographic knee OA, bilateral knee radiographic OA, and symptomatic knee OA in a cohort of individuals from a rural community in the southeastern United States, after adjusting for occupation and the primary risk factors for knee OA including age, BMI, gender, knee injury and occupational activity score. This is the first study to examine the role of a contextual measure of community SES in addition to individual level SES measures in association with knee OA. Our results extend findings noting the significant independent associations between individual and community SES measures with the frequency of self-reported arthritis [12] to radiographic and symptomatic knee OA. As noted in self-reported arthritis, we found both educational attainment and community household poverty rate were independently significantly associated with radiographic and symptomatic knee OA. In addition, this is the first study to examine the role of a possible community segregation variable, percentage of African American residents in a block group, and its association with knee OA. We found, though, that due to the substantial correlations between household poverty rate and our measure of segregation that there was no advantage to including both community contextual measures in our final models.

Compared to our previous study examining limited educational attainment and radiographic and symptomatic knee OA in the Johnston County OA Project [4], the data and approach in this study differ in several ways: (1) the participant sample was expanded about 37% by including new enrollees from the cohort enrichment from 2003 to 2004; (2) men and women were analyzed together since tests showed no significant effect modification by gender on the SES variables for any outcome (our previous stratification by gender was motivated by looking for effects of hormone replacement therapy); (3) occupational status was treated as a main, individual SES variable rather than as a potential confounding variable, and; (4) census block group household poverty rate was included as a main, community contextual SES variable.

Given the high prevalence of radiographic and symptomatic knee OA [1,21], it is important to understand the association of both individual level and community level SES measures with the disease. A number of behavioral and lifestyle factors have been discussed as possible mediators of associations between lower educational attainment and knee OA [4]. These include dietary factors [22-24] and psychological variables, such as depression, self-efficacy, and helplessness [25]. Much less has been explored in terms of community SES variables, and the work relating to contextual community variables in knee OA has focused more on disease management (for example, use of community resources for arthritis management) rather than the prevalence of disease [26,27]. The socioeconomic context of communities may affect characteristics of the physical environment all residents are exposed to regardless of their own socioeconomic position. This could have an impact on the availability of medical and recreational facilities, senior centers, and safe environments for engaging in physical activity [26]. These are all community resources that may be relevant and essential to an individual's management of their weight, an important risk factor for OA [26,28]. The socioeconomic context of a community could also influence an individual's exposure to environmental toxicants that may have an impact on OA [29]. A better understanding of these important contextual mediators could help national, state and local policy makers evaluate communities for resources that might help reduce the prevalence of chronic conditions that contribute the most to disability and reduced quality of life.

The primary strengths of our study are that we had measures of both radiographic and symptomatic knee OA and our data had a large representation of African Americans. Our study took place in a non-urban setting. We were also able to examine a measure of segregation in addition to household poverty as a contextual variable.

Our study is limited in that the data were collected in a single county, which limits its generalizability. We also did not have a measure of participant income to include as an individual SES measure, but we have shown in other studies that although income is probably the individual SES variable with the most significant impact on arthritis outcomes, educational attainment is still a very strong indicator of social position [30]. Our contextual measures of community SES, household poverty rate and the percentage of African Americans in a block group are fairly crude measures of a neighborhood or community. However, even using crude community SES measures from the Census, we noted independent significant associations between knee OA and both community poverty and educational attainment.

## Conclusions

In conclusion, in examining OA outcomes, it is important to consider both an individual's community and personal socioeconomic position in addition to other important demographic and lifestyle risk factors that are known to be associated with radiographic and symptomatic knee OA. Further research is needed to identify the specific attributes of a community that contribute to these associations. A better understanding of the role of community resources and environmental exposures would help policymakers and the public health community develop effective interventions and programs, and support improvements to the built environment targeted toward reducing knee OA.

## Abbreviations

AP: anterior-posterior; BMI: body mass index; KL: Kellgren-Lawrence; MAR: missing at random; MCAR: missing completely at random; MICE: multiple imputation by chained equations; NHANES-I: the first National Health and Nutrition Examination Survey; OA: osteoarthritis; PA: posterior-anterior; PSU: primary sampling unit; rOA: radiographic OA; sxOA: symptomatic osteoarthritis; SES: socioeconomic status

## Competing interests

The authors declare that they have no competing interests.

## Authors' contributions

LFC and JS contributed to study conception and design, participated in data analysis and interpretation, and in the drafting and reviewing of the manuscript. RC contributed to data analysis and interpretation, and in the drafting and reviewing of the manuscript. BS, TS, JR, RR and JMJ contributed to study design and interpretation, and reviewing of the manuscript. All authors read and approved the final manuscript.
